# Characterisation of antibiotic resistance, virulence, clonality and mortality in MRSA and MSSA bloodstream infections at a tertiary-level hospital in Hungary: a 6-year retrospective study

**DOI:** 10.1186/s12941-020-00357-z

**Published:** 2020-05-07

**Authors:** Andrea Horváth, Orsolya Dobay, Judit Sahin-Tóth, Emese Juhász, Júlia Pongrácz, Miklós Iván, Enikő Fazakas, Katalin Kristóf

**Affiliations:** 1 Institute of Medical Microbiology, Semmelweis University, Nagyvárad tér 4, 1089 Budapest, Hungary; 2 Institute of Laboratory Medicine, Semmelweis University, Budapest, Hungary

**Keywords:** *Staphylococcus aureus*, Bloodstream infection, MRSA, MSSA, Clonality

## Abstract

**Background:**

*Staphylococcus aureus* bloodstream infections (BSI) cause significant morbidity and mortality due to the frequent antibiotic resistance, toxin and adhesin production of the bacterium. These characteristics differ significantly in methicillin resistant (MRSA) and methicillin sensitive *S. aureus* (MSSA) and also among isolates of different MRSA clones, contributing to the outcome of *S. aureus* bacteraemia.

**Methods:**

In this study, all MRSA BSI isolates from Semmelweis University, Budapest, Hungary, isolated between 2011–2016 and the same number of matched MSSA (overall 306 isolates) were characterised in terms of antibiotic susceptibility, virulence genes, clonality and their association with all-cause 30-day mortality. Effect of patient related variables, such as age, gender and comorbidities were also investigated.

**Results:**

ST22-MRSA-IV and ST5-MRSA-II were the most prevalent clones in our study. SCC*mec* I isolates showed the highest resistance rates and SCC*mec* II carried most virulence genes. Infections caused by SCC*mec* IV isolates were associated with the highest mortality rate (42.2%), despite the similar comorbidity rates of the different patient groups. All-cause 30-day mortality was 39.9% in the MRSA and 30.7% in the MSSA group. Increased teicoplanin MIC was associated with high mortality rate. Resistance to ciprofloxacin, erythromycin and clindamycin was common in MRSA, whereas MSSA isolates were more sensitive to all antibiotics with the exception of doxycycline. All MRSA isolates were sensitive to glycopeptides and linezolid; resistance to rifampicin and sulfamethoxazole-trimethoprim was low. MRSA isolates carried more adhesion genes, superantigens were more frequent in MSSA. Panton-Valentine leukocidin was found in 2.3% of the isolates.

**Conclusion:**

This study provides insight into the clonal composition and associated mortality of BSI *S. aureus* isolates in Hungary. The results suggest that the outcome of the infection is determined by the antibiotic resistance, genotype of the bacterium, and patient-related factors; rather than the virulence factors carried by the bacteria.

## Background

Bloodstream infections (BSI) are the most severe form of *Staphylococcus aureus* infections [1]. Frequent antibiotic resistance, toxin and adhesin production of the bacterium result in significant morbidity and mortality.

Nevertheless, not all *S. aureus* isolates are the same. Antibiotic resistance and virulence of methicillin resistant (MRSA) and methicillin sensitive (MSSA) *S. aureus* differ significantly, contributing to the variable outcome of *S. aureus* BSI. Moreover, isolates of different MRSA clones also vary significantly in their antibiotic susceptibility, virulence and speed of replication, however, the impact of a specific clone on the clinical outcome of the infection is less studied.

*Staphylococcus aureus* express many cell-surface associated adhesins termed ‘microbial surface components recognizing adhesive matrix molecules’ (MSCRAMMs), allowing the bacterium to bind to extracellular matrix proteins (ECM) of the host, contributing to invasion and infection. Polysaccharide intercellular adhesin (PIA), also referred as poly N-acetylglucosamine (PNAG) mediates bacterial adhesion and is important part of staphylococcal biofilm. PIA is synthetized by N-acetylglucosamyl transferase, the product of *icaA* gene [2]. Collagen binding protein (CNA) has an important role in the pathogenesis of *S. aureus*, enhancing the adherence of the bacterium to connective tissue, and thus allow to cuase cause wound, skin and soft tissue infections [3]. Staphylococcal Protein A (SpA) is produced by the vast majority of clinical *S. aureus* strains and by binds to Fc and Fab domains of IgG antibodies, thus supresses immune response [4].

It is well**-**established that MRSA isolates are often multi-resistant towards antibiotics of different classes, while a significant proportion of MSSA strains are sensitive to non-β-lactam antibiotics. The virulence of the pathogen and the outcome of the infection are, however, difficult to compare between MRSA and MSSA isolates. Most studies report increased mortality rate in patients with MRSA infections [5]. Some other investigations debate this, suggesting that adjustment to confounding factors, such as comorbidities, age and severity of illness, and the delayed initiation of effective therapy may nullify the impact of resistance on outcome [6]. As toxin gene frequency may be as high in MSSA isolates as in MRSA, infections caused by MSSA should be taken seriously [7].

The genotype of the isolate may also have a role in the severity and outcome of the infection. In Hungary, during the 1990s the most prevalent *S. aureus* lineage was the ST239-MRSA-III (Hungarian/Brazilian) clone, replaced by the ST228-MRSA-I (South-German) and the ST5-MRSA-II (New York-Japan) clone from the beginning of the 2000s [8, 9]. An ESCMID survey on dominant clones of BSI *S. aureus* isolates in the European region in 2011 described ST22-EMRSA-15 being abundant in Hungary [10]. However, to our knowledge, this is the first comprehensive study describing antibiotic resistance, virulence factors and current clones of *S. aureus* BSI isolates from Hungary.

The objectives of this study were: [1] to compare antibiotic susceptibility, prevalence of virulence factors, genotype and mortality of patients with MRSA and MSSA strains from blood-stream infections over a 6-year period and [2] to gain insight into the *S. aureus* population currently causing bloodstream infection at our tertiary level health care clinic in Budapest, Hungary.

## Methods

### Strain collection

All non-duplicated blood stream infection (BSI) MRSA strains**—**isolated between January 2011 and December 2016, at the Institute of Laboratory Medicine, Semmelweis University, Budapest, Hungary**—**were included. Our laboratory serves a 2200-bed teaching hospital, with 135 000–140 000 inpatients admitted annually. Each year, the same number of MSSA BSI isolates, representing the same gender and age distribution of population and hospital wards were enrolled (from a much larger pool) to be compared to the MRSA strains. In total, 306 *S. aureus* BSI isolates (153 MRSA and 153 MSSA strains) were analysed. The isolates were non-epidemic strains. Patient data collected for each isolate included gender, age, comorbidities, current chemotherapy and steroid therapy, and all-cause 30-day mortality. Charlson comorbidity index was determined for each patient.

The statistical analysis of the results was carried out by Pearson’s Chi square test and Mann–Whitney U test using Microsoft Office Excel Analysis ToolPak. P-values less than 0.05 were considered statistically significant.

### Identification and antibiotic susceptibility testing

The identification of *S. aureus* strains was carried out by standard and MALDI-TOF MS analysis (Bruker Corporation, USA). Genotypic identification was based on the detection of *nucA*, *mecA and mecC* genes by PCR [11, 12]. The ATCC 33591 and ATCC BAA-2312 strains were applied as positive controls for the *mecA* and *mecC* PCR, respectively.

Antibiotic susceptibility to oxacillin, erythromycin, clindamycin, gentamicin, tobramycin, amikacin, doxycyline, sulfamethoxazole-trimethoprim, rifampicin, linezolid and ciprofloxacin was tested by disc diffusion method, and MRSA isolates were additionally tested for vancomycin and teicoplanin susceptibility by broth microdilution according to the European Committee of Antibiotic Susceptibility Testing (EUCAST) guidelines [13].

### Molecular analysis of virulence genes

Presence of the genes *hla, hlb, hlg, hlg*-*v, spa, lukS*-*PV/lukF*-*PV, icaA, cna, sea, seb, sec, tst, eta, etb* was detected by PCR [14–20]. For the detection of *sea, eta* and *etb*, we used newly designed primers (Table 1).

**Table 1:** Primers used for detection of *sea, eta* and *etb* genes in *S. aureus* strains

Gene	Primer sequence (5′-3′)	Annealing temperature (°C)	Amplicon size (bp)
*sea for*	TTATCAATGTGCGGGTGGTA	54	265
*sea rev*	CCTCTGAACCTTCCCATCAA		
*eta for*	AAAAACCATGCAAAAGCAGAA	54	372
*eta rev*	ACCTGCACCAAATGGTTCTT		
*etb for*	CAGCGCAGAAGAAATCAGAA	54	609
*etb rev*	CCGCCTTTACCACTGTGAAT		

### Genotyping

Pulsed field gel electrophoresis (PFGE) was performed after *Sma*I digestion for 151 MRSA and 153 MSSA strains according to a previously published method [21]. SCC*mec* typing was performed for all MRSA isolates by PCR as described previously [22, 23]. Multi locus sequence typing (MLST) was carried out on a subset of representative isolates of the most prevalent PFGE pulsotypes and SCC*mec* types identified in our study and MLST sequence types were assigned through the MLST database [24].

## Results

### Patient characteristics

Most of our BSI samples in the study period originated from patients admitted to the internal medicine unit (42.2%), intensive care unit (18.0%), haematology (15.7%), with less isolates recovered from cardiology (10.3%), surgery ward (6.5%), transplant clinic (3.9%) and pulmonology ward (2.9%) of the University.

The baseline characteristics and Charlson comorbidity index (CCI) of the patients are shown in Table 2. Chronic liver disease and chemotherapy was more frequent in MSSA patients, whereas more of MRSA patients had surgery in the previous 30 days or endocarditis, however, Charlson comorbidity index did not differ significantly in the two groups (Table 2).

**Table 2:** Characteristics of *S. aureus* BSI patients

	MSSA		MRSA		*P*
	n	%	n	%	
Diabetes	48	31.4	57	37.3	0.2785
Chronic liver disease	*33*	*21.6*	13	8.5	*0.0014*
Chronic kidney disease	26	17.0	21	13.7	0.4279
Solid tumor	39	25.5	25	16.3	0.0491
Haematology malignancy	20	13.1	15	9.8	0.3691
Chemotherapy	*21*	*13.7*	4	2.6	*0.0006*
Steroid treatment	14	9.2	9	5.9	0.2783
Surgery in previous 30 days	34	22.2	*54*	*35.3*	*0.0115*
Endocarditis	3	2.0	*12*	*7.8*	*0.0172*
Charlson comorbidity index (mean)	4.65		4.36		0.72634

The dominance of male gender among MRSA infections was statistically significant (61.4% males versus 38.6% females, p = 0.044). CCI was significantly higher in female patients (4.92 vs 4.24 in males, p = 0.0164).

Age range of the MRSA patients was 0–98 years (median = 68 years). MSSA patients were selected to match these figures as close as possible (0–94 years, median = 64 years).

### Antibiotic resistance of MRSA and MSSA strains

**Fig. 1:**
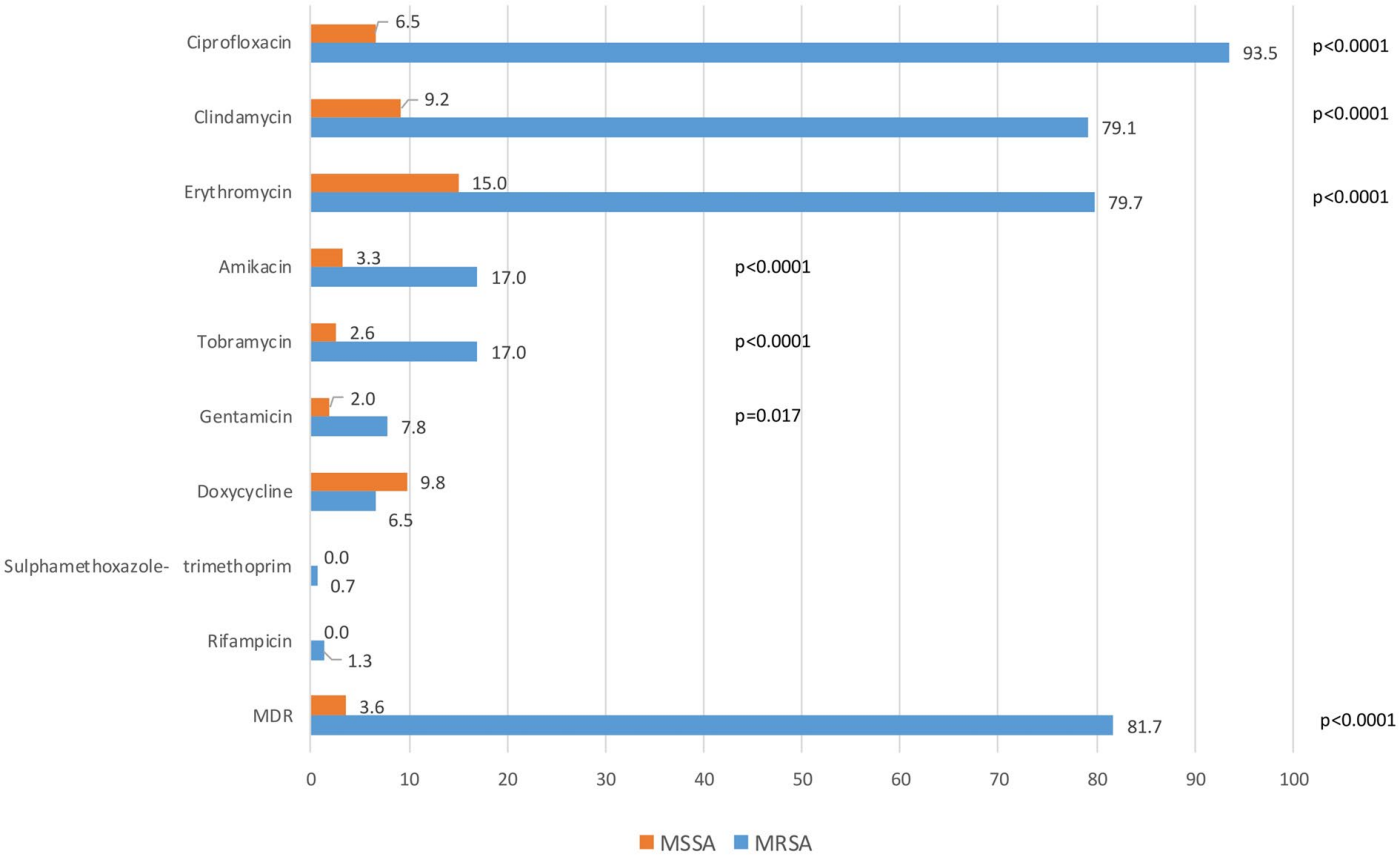
Antibiotic resistance of MRSA and MSSA isolates (Resistance rate (%) and multidrug resistance rate (MDR) (%))

Resistance rates of the MRSA isolates were significantly higher towards ciprofloxacin, erythromycin, clindamycin, amikacin, tobramycin and gentamicin compared to MSSA isolates. The resistance rates of MSSA isolates were the highest to erythromycin and doxycycline (Fig. 1). Almost all isolates were sensitive to sulfamethoxazole-trimethoprim and rifampicin.

The majority of MRSA isolates was multidrug-resistant (81.7%), i.e. resistant to at least three different antibiotic classes. Most prevalent resistance phenotype was resistance to β-lactams, erythromycin, clindamycin and ciprofloxacin. On the other hand, 75.8% of MSSA isolates were susceptible to all tested antibiotics and only 3.6% of them were multidrug-resistant (Fig. 1).

All MRSA and MSSA isolates were sensitive to vancomycin, teicoplanin and linezolid. Glycopeptide MICs ranged < 0.5–2 mg/L in each year. Vancomycin MIC was 2 mg/L in 7.8% of isolates. While in 2011–2012 less than 42% of the isolates had MIC ≥ 1 mg/L, in 2013–2015 more than 60% of the strains had MIC ≥ 1 mg/L. In 2016, their prevalence decreased to 51.7%. Vancomycin MIC_50_ values also increased from 0.5 mg/L in 2011–2012 to 1 mg/L in 2013–2016. Six point five percent of the isolates had teicoplanin MIC = 2 mg/L.

### Virulence factors of MRSA and MSSA isolates

Among the examined virulence genes, toxic shock syndrome toxin and exfoliative toxin A and B encoding genes (*tst, eta, etb*) were detected in 1.3% of all strains. *LukS*-*PV/lukF*-*PV* gene was found in 2.3% of the isolates (Table 3). Out of the 14 studied virulence genes, *cna, sea, ica* and *hlb* were significantly more prevalent in MRSA, whereas *tst*, *eta, sec* and *hlgv* were significantly more frequent in MSSA. Superantigens were more frequent in MSSA isolates, while adhesins were more frequent in MRSA isolates (Tables 2 and 3). *LukS*-*PV/lukF*-*PV* positivity rate was 3.3% and 1.3% in MRSA vs MSSA, respectively. The prevalence of this gene changed significantly during the 6 years of the study: in was 13% in MRSA isolates in 2011, but never exceeded 4% in the later years.

**Table 3:** Prevalence of virulence factors in MRSA and MSSA isolates

Virulence genes	MRSA		MSSA		All		p values
	n	%	n	%	n	**%**	
Superantigens							
tst	0	0.0	*4*	*2.6*	4	1.3	*0.044*
eta	0	0.0	*4*	*2.6*	4	1.3	*0.044*
etb	1	0.7	3	2.0	4	1.3	0.3141
sea	*30*	*19.6*	17	11.1	47	15.4	*0.0390*
seb	58	37.9	49	32.0	107	35.0	0.2806
sec	12	7.8	*25*	*16.3*	37	12.1	*0.0223*
Cytotoxins							
lukS-PV/lukF-PV	5	3.3	2	1.3	7	2.3	0.2513
hla	111	72.5	118	77.1	229	74.8	0.3564
hlb	*106*	*69.3*	75	49.0	181	59.2	*0.0003*
hlg	89	58.2	84	54.9	173	56.5	0.5642
hlg-v	31	20.3	*93*	*60.8*	124	40.5	*<* *0.0001*
Adhesins							
icaA	*122*	*79.7*	85	55.6	207	67.6	*<* *0.0001*
spa	150	98.0	152	99.3	302	98.7	0.3141
cna	*110*	*71.9*	45	29.4	155	50.7	*<* *0.0001*

Italic values indicate statistically significant associations. (p < 0.05)

MRSA strains carried a median of six virulence genes. The most frequent virulence type in MRSA was positivity for *hla, hlb, hlg, ica, spa, cna*, and *sea* or *seb* (11.1% and 14.4% of the isolates, respectively). Isolates were highly diverse; we identified 57 different virulence gene combinations in MRSA isolates. MSSA strains carried less virulence factors (median of 5). Most frequent virulence type in MSSA was *hla, hlb, hlg, hlgv, ica, spa* positivity.

### Clonality of the isolates

**Fig. 2:**
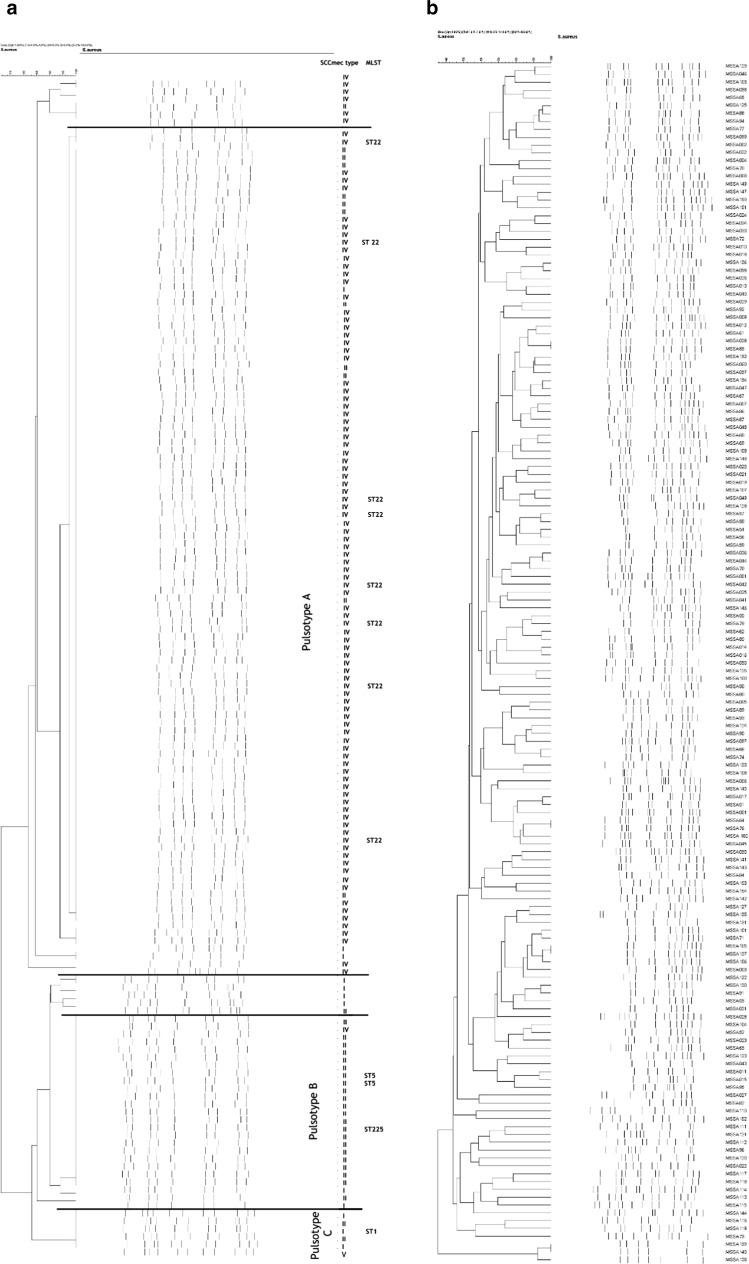
PFGE patterns of the MRSA strains (**a**) and the MSSA strains (**b**)

PFGE divided MRSA strains into 3 main pulsotypes, while MSSA strains proved to be much more diverse, no dominant clone could be identified (Fig. 2a, b).

The vast majority of the MRSA isolates in our study belonged to SCC*mec* type IV (66.7%). SCC*mec* II accounted for 23.5%, SCC*mec* I for 9.2% of the strains. One isolate belonged to SCC*mec* type V, whereas SCC*mec* types III and VI were not found. SCC*mec* type IV isolates were significantly more frequent in females (78.0% vs 59.6% in males, p = 0.0188).

MLST analysis was carried out on 12 representative MRSA isolates from the most frequent PFGE pulsotypes and SCC*mec* types, representing all 6 years of the study. All eight tested SCC*mec* IV, PFGE type A isolates belonged to the ST22 clone. Three SCC*mec* II, PFGE type B isolates were typed: two belonged to ST5 and one to ST225. Our representative SCC*mec* type I, pulsotype C isolate belonged to ST1.

Although SCC*mec* IV isolates dominated among the MRSA, these showed lower resistance to most antibiotics compared to SCC*mec* I and II isolates. Especially SCC*mec* I was associated with high resistance rates to aminoglycosides and doxycycline. Furthermore, the highest vancomycin MICs were observed also among these latter SCC*mec* types (Table 4).

**Table 4:** Antibiotic resistance rates and high vancomycin MIC in different SCC*mec* types

	SCC*mec* I n = 14		SCC*mec* II n = 36		SCC*mec* IV n = 102		MRSA all n = 153	
	R	%	R	%	R	%	R	%
Erythromycin	13	92.9	33	91.7	75	73.5	122	79.7
Clindamycin	13	92.9	32	88.9	75	73.5	121	79.1
Gentamicin	6	42.9	3	8.3	3	2.9	12	7.8
Tobramycin	7	50.0	12	33.3	7	6.9	26	17.0
Amikacin	7	50.0	12	33.3	7	6.9	26	17.0
Ciprofloxacin	11	78.6	35	97.2	97	95.1	143	93.5
Sulfamethoxazole-trimethoprim	0	0.0	0	0.0	1	1.0	1	0.7
Doxycycline	3	21.4	1	2.8	5	4.9	10	6.5
Rifampicin	1	7.1	0	0.0	1	1.0	2	1.3
Vancomycin MIC = 2 mg/L	3	21.4	4	11.1	4	3.9	10	6.5

SCC*mec* II isolates had the highest number of virulence genes. Panton-Valentine leukocidin was found only in SCC*mec* I and II isolates (Table 5). Interestingly, our single SCC*mec* V isolate did not carry any of the tested virulence factors.

**Table 5:** Prevalence of virulence genes in different SCC*mec* types

	SCC*mec* I n = 14		SCC*mec* II n = 36		SCC*mec* IV n = 102		MRSA all n = 153	
	R	%	R	%	R	%	R	%
tst	0	0.0	0	0	0	0	0	0%
eta	0	0.0	0	0	0	0	0	0%
etb	0	0.0	1	2.8	0	0	1	0.7%
sea	6	42.9	11	30.6	13	12.7	30	19.6%
seb	5	35.7	12	33.3	41	40.2	58	37.9%
sec	0	0.0	4	11.1	8	7.8	12	7.8%
pvl	2	14.3	3	8.3	0	0.0	5	3.3%
hla	10	71.4	29	80.6	72	70.6	111	72.5%
hlb	8	57.1	23	63.9	75	73.5	106	69.3%
hlg	5	35.7	22	61.1	62	60.8	89	58.2%
hlg-v	10	71.4	19	52.8	2	2.0	31	20.3%
icaA	11	78.6	28	77.8	83	81.4	122	79.7%
spa	14	100.0	36	100.0	100	98.0	150	98.0%
cna	5	35.7	12	33.3	93	91.2	110	71.9%
Median of n of virulence genes	5.5		7		6		6	

### Differences in mortality

Overall 30-day mortality was 35.3% in our BSI *S. aureus* cases, with higher rates in BSI cases caused by MRSA (39.9% vs 30.7% in MSSA, respectively, p < 0.0001), although CCI did not differ significantly in the 2 groups.

Females had significantly higher CCI than males, this can attribute to the higher mortality rate in this group (38.7% vs 33.2% of fatalities in females and males, respectively (p < 0.001)). Mortality increased with age: it was 20.0% in age group 0–49 years, 28.0% in 50–64 years, 40.2% in 65–79 years and 55.8% in patients older than 80 years.

Higher vancomycin MIC did not influence mortality risk. On the other hand, although we have found only 10 isolates with teicoplanin MIC of 2 mg/L, mortality was 70% in this group.

The number of carried virulence genes, the presence of specific virulence factors and antibiotic resistance to other drugs besides glycopeptides did not influence mortality.

Association of genotype, CCI and mortality are shown in Table 6. Interestingly, patients infected with SCC*mec* IV isolates had higher mortality, than patients infected with SCC*mec* I and II MRSA strains, however, these differences were not statistically significant. CCI of SCC*mec* I group and SCC*mec* II group do not differ significantly when compared to CCI of SCC*mec* IV group.

**Table 6:** Association of *S. aureus* genotype, CCI and mortality rate

	MRSA-SCC*mec* I	MRSA-SCC*mec* II	MRSA-SCC*mec* IV	MSSA
Charlson comorbidity index	4.29	3.97	4.46	4.65
Mortality rate (%)	28.6	36.1	42.2	30.7

## Discussion

### Differences between MRSA and MSSA

*Staphylococcus aureus* is a significant and prevalent pathogen, however, the importance of methicillin resistance in the virulence of the bacterium and in the outcome of the infection is still not completely clear. According to a recent study from the USA, MRSA bacteraemia is associated with a higher risk of readmission for bacteraemia recurrence, increased mortality, and longer hospitalization [25]. Most studies support the concept that MRSA BSI is associated with poorer outcome [5], while some other researchers debate this, and report mortality comparable to that in MSSA BSI [6, 26]. Some studies even suggest that MSSA strains may cause more severe infections, which might be related to higher prevalence of virulence genes in MSSA or to the greater fitness cost associated with SCC*mec* cassettes in MRSA [27]. In our study, we observed higher mortality rates in patients with MRSA infections than those with MSSA. Female gender, older age, infection with SCC*mec* IV isolate and teicoplanin MIC = 2 mg/L were additional risk factors for mortality.

Higher antibiotic resistance of MRSA isolates may be an explanation for high mortality rates, as inappropriate empirical antibiotic therapy is described to be more frequent in patients with MRSA bacteraemia [28]. In our study, we found significantly higher resistance rates to several antibiotics and also more frequent multidrug resistance rate in MRSA than in MSSA as well.

In addition, we found different patterns of virulence genes in MRSA and MSSA isolates. Particularly adhesion factors (*cna* and *ica*) were significantly more prevalent in MRSA, meanwhile genes encoding for superantigens (especially *sea* and *eta and tst*) were more prevalent in MSSA isolates (Table 3). We found a low overall prevalence of *pvl.* On average, MRSA isolates carried more virulence genes than MSSA isolates. However, the number of the carried virulence genes or the presence of specific virulence genes did not influence 30-day all-cause mortality. Our findings suggest that the outcome of the infection is related to the antibiotic resistance and clonality of the bacterium and to patient-related factors, such as age and gender, rather than the virulence factors of the bacteria.

### Antibiotic susceptibility

#### MRSA rates

In our laboratory, the MRSA rate among BSI *S. aureus* isolates varied between 27.5% and 40.7% during the investigated 6-years period. Similarly to a number of countries worldwide, MRSA prevalence decreased in Hungary in the recent years [29]. According to the surveillance data from the National Public Health Institute of Hungary, the proportion of MRSA strains among invasive *S. aureus* samples in Hungary increased from 20.2% to 30.1% between 2005 and 2010 (p < 0.001), then decreased to 24.2% by 2012 (p < 0.001). Since 2012, the prevalence of oxacillin resistance remained stable around 24% (data published in Hungarian) [30]. As our laboratory serves mostly university clinics, different patient population and increased disease severity may be responsible for the higher MRSA rates compared to the national average.

#### Glycopeptide susceptibility

The gradual increase of the number of MRSA isolates with high glycopeptide MIC values, referred as ‘vancomycin MIC creep’ in the literature is controversial; many studies report an increase of MICs, while others did not confirm these findings [31]. In our study, all MRSA isolates were sensitive to glycopeptides, however, vancomycin MIC seemed to creep higher from 2011 until 2015, with a slight decrease in 2016. The possibility of gradual increase in vancomycin MIC requires special attention, as it might lead to the development of resistant strains, and poorer clinical outcome was reported in patients infected with isolates exhibiting higher glycopeptide MIC values [31]. In our study, elevated vancomycin MIC was not associated with increased 30-day mortality, but patients with teicoplanin MIC = 2 mg/L had higher mortality than those with low teicoplanin MIC values.

Rifampicin resistance was very low in MRSA in our study, and none of the MSSA isolates were resistant to this drug, similar to data from other European countries [29]. Almost all of our isolates have retained susceptibility towards sulfamethoxazole-trimethoprim, in concordance with reports from Europe and other locations worldwide [32, 33].

### Virulence

The prevalence of virulence genes in BSI *S. aureus* isolates varies highly according to geographical region and patient population.

Several studies have found low prevalence of PVL in *S. aureus* isolates from BSIs, and described it to be more closely associated with skin and soft tissue infections [27, 34]. However, for instance, *pvl* in BSI was more prevalent in Romania (22.4%) [35]. In our study, we found low *pvl* rate (2.3% for all samples). In a recent study on BSI MSSA, decreasing prevalence of *pvl* and other virulence genes during recent years was observed [27]. This is in concordance with our findings in MRSA isolates: in 2011, 13.0% of our MRSA isolates were positive for *pvl*, however, in the following years *pvl* frequency never exceeded 4% in our MRSA isolates. *Tst* was found exclusively in MSSA. Number of carried virulence genes and presence of specific virulence factors did not influence the outcome of the infection.

### Clonality

It is well demonstrated that successful *S. aureus* clones are invading and replacing their competitors, changing the clonal map over time. In North and South America, and in Japan, USA 300 MRSA clone is becoming more prevalent, while in Europe and Asia ST239 Hungarian-Brazilian strains are being replaced by ST22-MRSA-IV (also known as EMRSA-15) [36]. According to a South-German study from 2016, this strain appeared in 2001 and became rapidly more common in their samples, accounting for nearly 80% of the MRSA strains in 2013 [35]. It was described as the most prevalent sequence type in NICU patient in Italy [37]. It has been causing nosocomial infections in the UK and in Ireland since the beginning of the 2000s [38] and was also described outside Europe, for example in Kuwait [39]. However, there are considerable differences in antibiotic susceptibility and virulence factors between variants of ST22-MRSA-IV clone. For example, UK-EMRSA-15/”Middle Eastern Variant” is generally susceptible to antibiotics and is characterized by the presence of *tst1* gene, whereas our isolates completely lack this gene [37]. Another variant of ST22-MRSA-IV clone is positive for PVL [35], however, all of our SCC*mec* IV strains were PVL negative. SCC*mec* IV was previously considered as a usually community-acquired MRSA, however, it became widespread and successful in hospital settings, too [40]. In Hungary, ST22-MRSA-IV has been the most frequently described clone among BSI isolates since 2008, its prevalence was nearly 60% in 2013, however, its prevalence is decreasing in the recent years (personal communication from Ákos Tóth, National Public Health Center of Hungary). In our study, the majority (66.7%) of the BSI MRSA isolates belonged to the ST22-SCC*mec* IV type.

Our SCC*mec* II isolates belonged to clonal complex 5 (CC5). ST5-MRSA-II and its MLST single locus variant, ST225-MRSA-II were both found. ST5-MRSA-II (New York –Japan or Rhine-Hesse clone) has high worldwide prevalence [40]. It was the most prevalent MRSA type in the 2000s in Hungary, until its replacement by EMRSA-15 [9]. A representative of our PFGE type C isolates belonged to ST1-MRSA-I, a non-epidemic clone, which was found in a low number of patients, for example, in Croatia and in Italy [41, 42].

In accordance with other studies, we have found high genotypic diversity in MSSA strains, no predominant clone was identified among those isolates (Fig. 2b) [27, 43].

#### Role of SCCmec in the resistance, virulence and mortality of infected patients

In our study, SSC*mec* I and II isolates were associated with the highest rates of antibiotic resistance, while SCC*mec* IV was associated with low resistance, except for ciprofloxacin (Table 4). According to the literature, SCC*mec* types I-II-III are more likely to exhibit high glycopeptide MIC values and vancomycin hetero-resistance, than SCC*mec* IV isolates [44], our findings are in concordance with this (Table 4).

SCC*mec* II isolates carried the highest number of virulence genes. All our *pvl* positive MRSA isolates belonged to SCC*mec* type I or II (Table 5). Earlier studies had associated Panton-Valentine leukocidin positivity with CA-MRSA, but later on *pvl* genes emerged in various MRSA clones via PVL bacteriophages [45].

Although SCC*mec* type I isolates had higher resistance rates to antibiotics and SCC*mec* type II strains had the most virulence genes, interestingly, infections caused by SCC*mec* type IV isolates had the highest mortality, whereas their CCI value did not differ significantly (Table 6). As described by Recker et al., bacterial phenotype and genotype are highly predictive for adverse infection outcome, and have stronger impact on mortality than other factors, such as patient age, gender or comorbidities [46]. Our findings support that the genotype of the bacterium has a major influence on the outcome of the infection. This underlines the importance of having up-to-date knowledge on the clonal types circulating at a given location.

Our study has the following limitations: it focuses on strains originating from a single centre, only a subset of the isolates was analysed by MLST, severity of the illness at presentation and antibiotic treatment of the patients were not analysed.

## Conclusions

In conclusion, antibiotic resistance and virulence of MRSA and MSSA isolates differ significantly. In our population, higher 30-day mortality was associated with BSI caused by MRSA and by strains with high teicoplanin MIC value. ST22-MRSA-IV was the dominant clone in our samples, and caused higher mortality than ST5-MRSA-II and ST1-MRSA-I strains. MSSA isolates had significantly lower antibiotic resistance rates and somewhat lower virulence gene prevalence than MRSA isolates. However, 30-day mortality of patients with MSSA BSI was still high, thus MSSA infections should be treated with appropriate care.

*Staphylococcus aureus* BSI continues to present a major clinical challenge. Further studies are required to gain a better understanding of differences in the antibiotic resistance, virulence and genotype of the bacterium and the impact of these factors on disease outcome.

## Data Availability

None.
